# Detection of Amyloid-β(1–42) Aggregation With a Nanostructured Electrochemical Sandwich Immunoassay Biosensor

**DOI:** 10.3389/fbioe.2022.853947

**Published:** 2022-03-16

**Authors:** Bing-Yu Wang, Bien-Chen Gu, Gou-Jen Wang, Yuan-Han Yang, Chia-Che Wu

**Affiliations:** ^1^ Department of Mechanical Engineering, National Chung Hsing University, Taichung, Taiwan; ^2^ Graduate Institute of Biomedical Engineering, National Chung Hsing University, Taichung, Taiwan; ^3^ Department of and Master’s Program in Neurology, Faculty of Medicine, Kaohsiung Medical University, Kaohsiung, Taiwan; ^4^ Department of Neurology, Kaohsiung Medical University Hospital, Kaohsiung Medical University, Kaohsiung, Taiwan; ^5^ Department of Neurology, Kaohsiung Municipal Ta-Tung Hospital, Kaohsiung Medical University, Kaohsiung, Taiwan; ^6^ Innovation and Development Center of Sustainable Agriculture (IDCSA), National Chung Hsing University, Taichung, Taiwan; ^7^ Smart Sustainable New Agriculture Research Center (SMARTer), Taichung, Taiwan

**Keywords:** Alzheimer disease (AD), amyloid-β(1–42) peptide, aggregation, oligomers, electrochemical impedance spectroscopy(EIS), nanostructured biosensor

## Abstract

Amyloid-β(1–42) [Aβ(1–42)] oligomer accumulations are associated with physiologic alterations in the brains of individuals with Alzheimer’s disease. In this study, we demonstrate that a nanostructured gold electrode with deposited gold nanoparticles, induced via electrochemical impedance spectroscopy (EIS), may be used as an Aβ(1–42) conformation biosensor for the detection of Alzheimer’s disease. Monoclonal antibodies (12F4) were immobilized on self-assembled monolayers of the electrochemical sandwich immunoassay biosensor to capture Aβ(1–42) monomers and oligomers. Western blot and fluorescence microscopy analyses were performed to confirm the presence of Aβ(1–42) monomers and oligomers. EIS analysis with an equivalent circuit model was used to determine the concentrations of different Aβ(1–42) conformations in this study. We identified conformations of Aβ(1–42) monomers and Aβ(1–42) oligomers using probe antibodies (12F4) by employing EIS. ${\text{R}}_{\text{Aβ}\left(1-42\right)}$ indicates the sum resistance of impedance measured during Aβ(1–42) immobilization. $\Delta {\text{R}}_{12\text{F}4}$ refers to the concentration of probe antibody (12F4) binding with Aβ(1–42). The concentration of Aβ(1–42) oligomer was defined as the percentage of Aβ(1–42) aggregation ${\text{R}}_{12\text{F}4}/{\text{R}}_{\text{Aβ}\left(1-42\right)}$ . The experimental results show that the biosensor has high selectivity to differentiate Aβ(1–40) and Aβ(1–42) monomers and Aβ(1–42) oligomers and that it can detect Aβ(1–42) oligomer accurately. The linear detection range for Aβ(1–42) oligomers was between 10 pg/ml and 100 ng/ml. The limit of detection was estimated to be 113 fg/ml.

## 1 Introduction

Alzheimer’s disease (AD) is the most common cause of dementia and can lead to severe memory loss and cognitive decline. Furthermore, patients with AD may lose their ability to live independently (Hendrie et al., 2006). AD predominantly affects the elderly and has become a serious societal issue. Most families living with AD confront isolation, unpredictability, fear, fatigue, and overwhelming loss of control (Gwyther, 1998). There is no known cure for AD, but some medications and alternative treatments exist with the aim of easing symptoms and delaying the progression of AD. Therefore, early diagnosis of AD is of vital importance to prevent and delay the progression of the disease (Cummings, 2011; Zhou et al., 2016).

Amyloid-β (Aβ), which is generated by β- and *d*-secretases (proteolytic enzymes) from amyloid precursor protein, is a well-known biomarker for AD (Selkoe, 1999; Kirkitadze et al., 2002; Schupf et al., 2008; Diba et al., 2017). AD is caused by the accumulation of insoluble amyloid plaques in the brain. Aβ(1–40) and Aβ(1–42) are the major components of amyloid plaques and are toxic to ischemic neuronal cells; they can be detected in human cerebrospinal fluid (CSF) and blood plasma (Bacskai et al., 2001; Parnetti et al., 2006; Giedraitis et al., 2007; Bird, 2008). The abnormal accumulation mechanism of amyloid plaques is associated with the secondary conformation of Aβ monomers caused by self-aggregation, which involves dimer, oligomer, fibril, and fibrillar aggregates. Recent studies have indicated that Aβ(1–42) is more hydrophobic than Aβ(1–40) and aggregates into fibrils at a markedly faster rate. The Aβ(1–42) oligomer is the most toxic form of Aβ and has the ability to disrupt membrane functions, thereby inducing neuronal damage (Levine, 2004; Takahashi et al., 2004; Islam et al., 2011). Aβ(1–42) is considered a reliable molecular biomarker for the diagnosis of AD (Lashuel et al., 2002; Hampel et al., 2010; Pluta et al., 2013; Chen et al., 2017). According to a study (Kravenska et al., 2020), the Aβ(1–42) monomers and fibrils might also contribute to AD progression. Thus, a sensor with high sensitivity and specificity to differentiate Aβ(1–42) monomer, oligomer, and fibril is needed for the early diagnosis of AD (Xing et al., 2017; You et al., 2020).

Many studies have developed methods for the detection of Aβ(1–42). Cullen, V. C. et al. used a modified INNOTEST^®^ Aβ42 ELISA kit to detect for the presence of Aβ(1–42) in CSF, and the concentration range of detection was 375–4,500 pg/ml (Cullen et al., 2012). M. Ammar et al. developed a surface-modified silicon wafer immunoassay with carboxylated alkyltrichlorosilane. The modified immunoassay can be self-assembled, and antibodies for Aβ(1–42) were utilized in a fluorescence-based sandwich assay. The limit of detection (LOD) was 300 ng/ml (Ammar et al., 2013). Paola Gagni et al. presented a highly sensitive immunoassay based on label/label-free Si/SiO_2_ substrates on a microarray platform to detect Aβ(1–42). The results indicated that the CoV-12F4 antibody had a good selectivity and sensitivity for the detection of Aβ(1–42) compared with other antibodies and the LOD was 73 pg/ml (Gagni et al., 2013). Ning Xia et al. used silver nanoparticles as the redox reporters and amyloid-β oligomers (AβOs)-specific peptide PrP(95–110) as the receptor to detect the AβOs using linear-sweep voltammetry. The biosensor had a detection limit of 8 pM and linear range of 20 pM–100 nM (Xia et al., 2016).

In recent times, electrochemistry has become a commonly used biological detection technique because it exhibits high sensitivity, reliability, and rapid detection times and does not require labels (Wang, 2005; Grieshaber et al., 2008; Rushworth et al., 2014). EIS involves the application of a small excitation signal to observe charge transfer at the electrode–electrolyte interface (Bard and Faulkner, 1980). A self-assembled monolayer (SAM) modified on the electrode for biomarker capture may represent a promising alternative to conventional immunoassay techniques (Chaki and Vijayamohanan, 2002; Love et al., 2005). Following EIS measurements, Hung et al. proposed that lipoic acid induces the self-assembly of Aβ (Hung et al., 2013). Veloso et al. employed EIS to detect the Aβ(1–42) aggregation process using anti-fibril and anti-oligomer antibodies, which were covalent with SAMs on Au electrodes (Veloso et al., 2014). Hien T. Ngoc Le et al. developed a chain-shaped electrode to detect Aβ(1–42) peptide by EIS. It had a linear range of 10^−3^–10^3^ ng/ml and detection limit of 100 pg/ml (Ngoc Le et al., 2019). Yuting Zhang et al. developed an aptasensor using an ssDNA aptamer as receptors to capture AβOs and monitored changes in the charge transfer resistance of redox probes using EIS. The proposed aptasensor exhibited a linear concentration detection range from 0.1 to 500 nM and detection limit of 0.03 nM (Zhang et al., 2019). Gopal Palla et al. presented a sensor with 4,40-thiobisbenzenethiol self-assembled monolayer on a clean gold surface followed by the covalent entrapment of gold nanoparticles for sensing Aβ(1–42). It had a detection limit of 0.64 pM and linear range of 0.5–4 pM (Palla et al., 2021). Pankaj D. Mehta et al. revealed that in patients with AD, the concentrations of Aβ(1–40) (100–770 pg/ml) and Aβ(1–42) (25–880 pg/ml) in the plasma were increased compared with healthy individuals (Mehta et al., 2000). L. Zhou et al. also indicated that levels of plasma Aβ(1–42) oligomers in patients with AD (642.5 ng/ml) were higher than in healthy individuals (Zhou et al., 2012). However, the above studies lacked selectivity between Aβ(1–40) and Aβ(1–42) monomers and Aβ(1–42) oligomers, which can alter AD diagnosis. Therefore, detection methods with a high selectivity and sensitivity for determining and differentiating between the concentration levels of Aβ(1–40) and Aβ(1–42) monomers and Aβ(1–42) oligomers are needed for accurate AD diagnosis.

To detect low concentrations of biomarkers, nanomaterials, and nanostructures were used to develop the biosensor. Tsia et al. demonstrated that an anodic aluminum oxide (AAO) nanostructure sputtered by Au on AAO film with deposited GNPs may be used as a template to increase biosensors’ abilities to capture the dust mite antigen Der p2. The LOD for this performed EIS analysis was 1 pg/ml (Tsai et al., 2011; Chin et al., 2013). To increase biosensor reproducibility, Chen et al. proposed novel nanostructure fabrications. The nano mold was made from AAO. Nano molds can replicate nanostructures in an identical fashion on polycarbonate (PC) by hot embossing. The LOD using a three-dimensional (3D) structure on a PC electrode with deposited GNPs could reach 100 fg/ml (Chen and Wang, 2012).

In this study, we demonstrate that a 3D nanostructure biosensor can differentiate Aβ(1–40), Aβ(1–42) monomers, and Aβ(1–42) oligomers, and detect low concentrations of Aβ(1–42) monomers and oligomers. To create a biosensor for Aβ capture, a thin Au film was used, and GNPs were uniformly deposited on the nano-hemisphere array PC substrate; monoclonal antibodies were then immobilized on the PC substrate. Following this process, EIS analysis was performed to determine the impedance of the biosensor. An electrochemical sandwich assay (capture antibody–antigen–probe antibody immunoassay) was also developed. The Aβ(1–42) monomer has one binding site that can be bound by an antibody. Comparatively, the Aβ(1–42) oligomer has one additional, residual binding site that can be bound by many antibodies, which may increase impedance. The difference in impedance between Aβ(1–42) and probe antibodies defines the concentration of Aβ(1–42) oligomer. Western blot and fluorescence-based sandwich assays were performed to verify the specificity of the Aβ(1–42) antigen and antibodies.

## 2 Materials and Methods

### 2.1 Chemicals

11-mercaptoundecanoic acid (MUA), 2-(N-morpholino) ethanesulfonic acid (MES), serum-coloring agents, protein agents, trifluoroacetic acid, hexafluoro-2-propanol (HFIP), rabbit anti-mouse IgG/FITC, and bovine serum albumin (BSA) were obtained from Sigma-Aldrich. Hydroxysuccinimide (NHS) and 1-ethyl-3-(3-dimethylami-nopropyl) carbodiimide hydrochloride (EDC) were purchased from Acros-Organics. K_3_[Fe(CN)_6_]·3H_2_O and K_4_[Fe(CN)_6_] were obtained from SHOWA Inc. 10X Phosphate buffered saline (PBS) buffer was purchased from GeneMark Inc. LC5800 pre-stained protein, Aβ(1–40) antigens, and Aβ(1–42) antigens were purchased from ENZO Life Science. Aβ(1–42) monoclonal antibodies (12F4), monoclonal antibodies (4G8), and immunoglobulin G (IgG) were obtained from NOVUS Inc. All chemicals were used without further purification.

### 2.2 Preparation of Aβ(1–42) Monomers and Oligomers

Aβ(1–42) monomers and oligomers were prepared in accordance with previously reported (Klaver et al., 2010; 2011) methodologies with slight modifications. Aβ(1–42) powder (1 mg) was dissolved in a solution of 0.5 ml trifluoroacetic acid (TFA) and 0.5 ml hexafluoro-2-propanol (HFIP). The solution was then aliquoted into an Eppendorf tube (100 µL/tube), dried for 24 h at room temperature in the fume hood, and then stored at −20°C for further use. To produce the Aβ(1–42) monomer, 1 ml TFA acid (pH = 3) was added to the stored Eppendorf tube, vortexed for 1 min, and then placed on ice for 30 min. Finally, the Aβ(1–42) monomer acid solution was passed through a 0.2 −μ m filter and diluted with PBS to generate staggered standard concentrations. To produce Aβ(1–42) oligomers, 8 µL of 1% NH_4_OH was added to the stored Eppendorf tube and vortexed for 1 min. Following this, the solution was sonicated in a water bath for 5 min, incubated at room temperature for 1 h, and then diluted in PBS (0.01 M, pH 7.4, with 0.02% azide) to generate staggered standard concentrations for further experimentation. Solutions were either used immediately or stored at 4° C for up to 3 days.

### 2.3 Fabrication of the Nanostructured Biosensor

**FIGURE 1:**
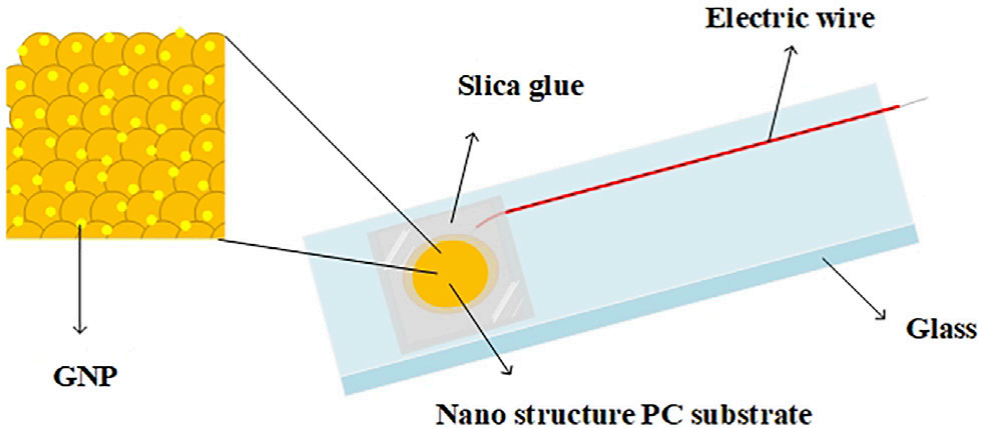
Schematic of the nanostructured biosensor, developed by sputtering a thin Au film on the 3D nanostructure PC substrate. AB and silica glue, functioning as insulators, were used to define a working area with a 5.5-mm diameter. Wire *via* contact with the Au film acted as a conductor for electrical signals.

A schematic illustration of the nanostructured biosensor developed in this study is shown in Figure 1. The nanostructured biosensor was developed by sputtering a thin Au film on the 3D nanostructure PC substrate. Using electrochemical methods, gold nanoparticles were deposited on the thin gold film in a uniform manner. The nanostructured biosensor was created in accordance with previously published research (Chen and Wang, 2012). First, an AAO membrane was prepared via an anodizing process via etching with phosphoric acid, producing a uniform hemisphere structure with a 400-nm diameter and a 75-nm height. The 3D nanostructure on the nickel mold was transferred from the AAO membrane. The nickel mold replicated the 3D nanostructure on the PC template by hot embossing. Au film (30 nm) was deposited on the 3D nanostructure PC substrate via radio frequency magnetron sputter. The PC substrate was then placed in a furnace set at 100°C for 90 min. Following this, an annealing procedure was performed to increase the homogeneity of the Au film. Finally, the PC substrate was used as the working electrode, the Pt plate as the counter electrode, the Ag/AgCl/3 M KCl as the reference electrode, and ${\text{HAuCl}}_{4}$ as the electrolyte in the electrochemical cell. A 0.7-V direct current (DC) electrical potential was applied for 180 s at room temperature to ensure the deposition of GNPs on the thin Au film. Each of GNP’s diameter was 10–15 nm. To ensure all chemical buffers remained in the reaction area of the biosensor, AB glue and silica glue, functioning as the insulators, were used to define a working area with a 5.5-mm diameter. Wire *via* contact with the Au film acted as a conductor for electrical signals.

### 2.4 Immobilization for Sensing Aβ(1–40) and Aβ(1–42)

**FIGURE 2:**
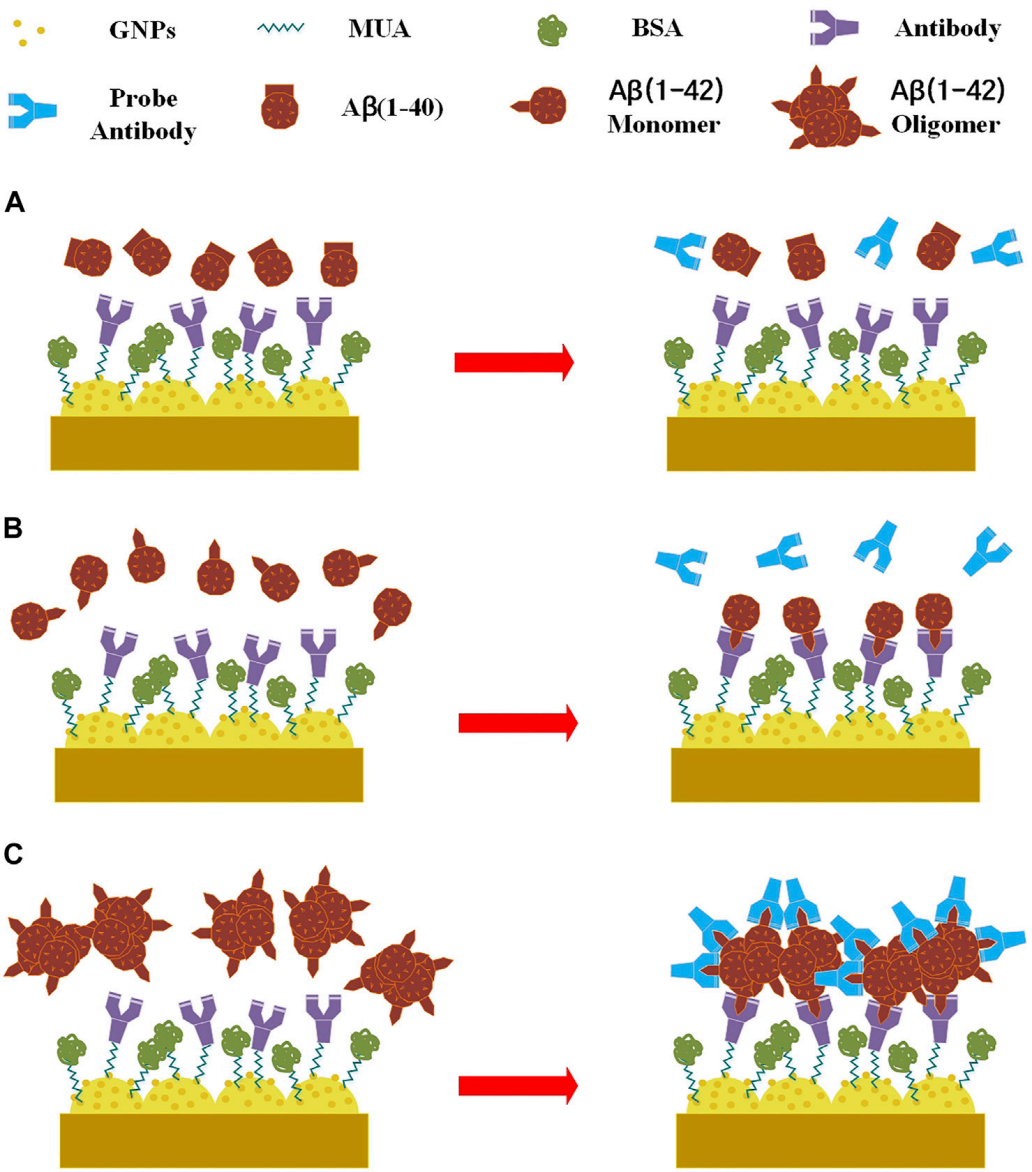
Schematic of immobilization, **(A)** Aβ (1–40) antigens do not bind to antibodies. **(B)** Aβ (1–42) monomers bind to antibodies, but the probe antibodies do not bind to Aβ (1–42) monomers. **(C)** Aβ (1–42) oligomers bind to antibodies, and the probe antibodies bind to residual binding sites of Aβ (1–42) oligomers.

The surface of the nanostructured biosensor was modified with a SAM to provide the site of immobilization for sensing Aβ(1–42) monomers and oligomers. The following steps were performed: 1) the surface of the biosensor was washed by successive immersion in acetone, ethanol, and deionized water, followed by ultrasonic shaking for 5 min 2) Droplets of MUA solution (10 mM, 20 μl) were successively administered to the biosensor for 30 min in an incubator set at 20% relative humidity and 37°C. Resultantly, alkanethiols self-assembled on the gold film. 3) The biosensor was cleaned with 99.5% ethanol and dried with nitrogen gas. 4) An EDC, NHS, and MES solution (30 µl) with a molar ratio of 1:15:7.5 (2 mM:30 mM:5 mM, respectively) was incubated on the MUA-modified layer of the biosensor for 1 h at 25° C. EDC/NHS was activated by the MUA carboxyl group. The biosensor was then washed three times with 1 ml double-distilled water. 5) The 30 µl immobilizing buffer containing monoclonal antibodies (12F4) ( 1ug/ml ) was then administered to the biosensor in an incubator set at 53% relative humidity and 25°C. When applied to the biosensor, the antibodies (12F4) substituted N-terms with N-hydroxysuccinimide. The biosensor was then washed three times with 1 ml double-distilled water. 6) A 30 µl 1% BSA solution was administered to the biosensor to prevent non-specific adsorption, which was then incubated for 1 h. The biosensor was then washed three times with 1µlml double-distilled water. 7) In order to perform capture of antigen [Aβ(1–40), Aβ(1–42) monomer and oligomer] by 12F4 antibodies, a 30-µL antigen buffer solution was applied to the biosensor, which was then incubated for 1 h. The residue of non-binding antigen was washed using 2 ml PBS and dried using nitrogen gas. 8) To allow probe 12F4 antibodies to bind to the residual binding sites of Aβ(1–42) oligomers, a 30 µL antibody (12F4) solution was again applied to the biosensor, which was then incubated for an additional 1 h. Finally, the 12F4 antibodies that had not conjugated with Aβ(1–42) oligomers were washed away using 2 ml PBS and dried using nitrogen gas. Figure 2 shows the schematic of immobilization. Aβ (1–40) antigens do not bind to antibodies. Aβ (1–42) monomers bind to antibodies, but the probe antibodies do not bind to Aβ (1–42) monomers. Aβ (1–42) oligomers bind to antibodies, and the probe antibodies bind to residual binding sites of Aβ (1–42) oligomers.

### 2.5 Preparation of Western Blot and Fluorescence Assays

Western blotting and fluorescence assays were performed to verify the prepared Aβ(1–42) monomers and oligomers. Polyacrylamide gels were formed from the polymerization of many compounds, including running buffer sodium dodecyl sulfate, pierce ammonium persulfate, and tetramethylethylenediamine. The 2-µL low molecular weight marker and the 25-µL samples were loaded in each well, and the gel was run for 2 h at 100 V. The electrophorezed proteins were then transferred to polyvinylidene difluoride (PVDF) membranes. Following this, membranes were incubated with mouse anti-Aβ(1–42) monoclonal antibodies (12F4) in 10 ml 2% blocking solution overnight at 4°C. After Tween-Tris-buffered saline (TTBS) washing, images of the gels were captured in a darkroom. For fluorescence assay, following the method in Section 2.4 [step (7)], a 30-µL rabbit anti-mouse IgG/FITC solution conjugated with Aβ(1–42) was applied to the biosensor, which was then incubated for an additional 2 h. The surface was rinsed three times with PBS containing Tween-20. The absorbance was measured at a wavelength of 450 nm, and fluorescent intensity was measured using a fluorescence microscopy.

### 2.6 Electrochemical Analysis

**FIGURE 3:**
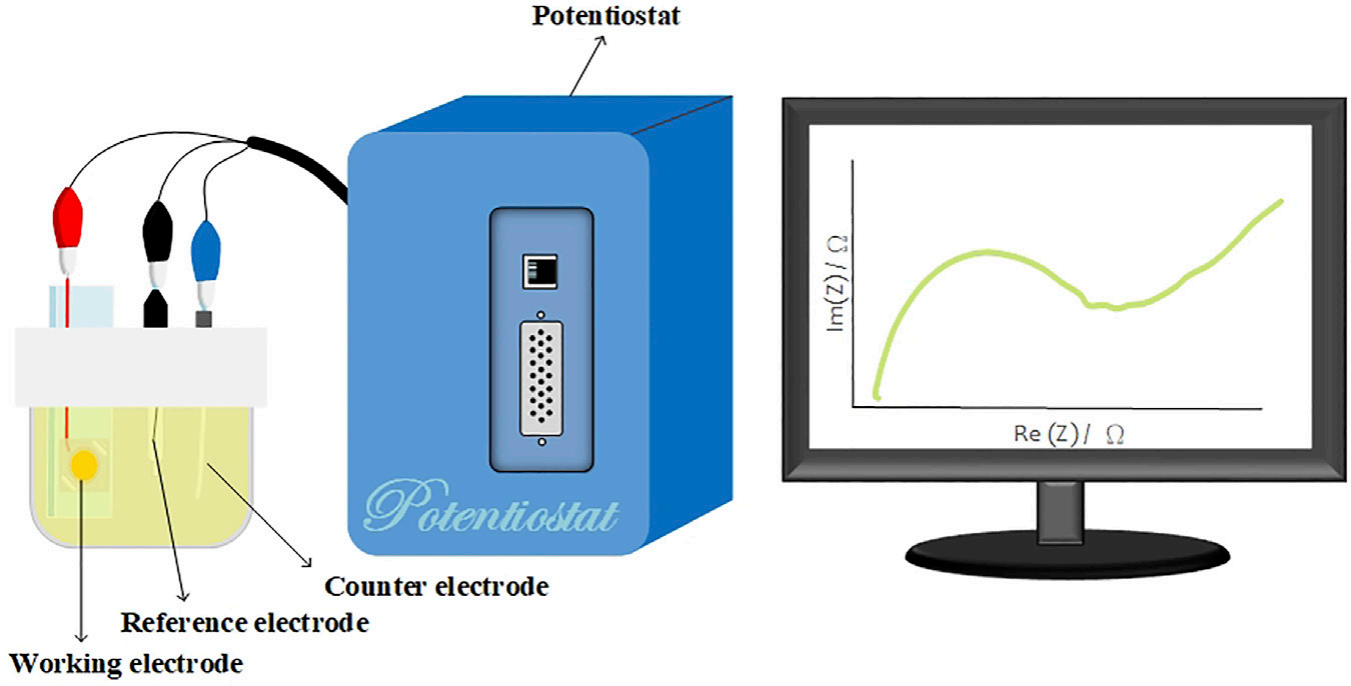
Schematic of the experimental setup. The surface-modified nanostructured biosensor served as the working electrode, and the Pt film and Ag/AgCl/3 M KCl functioned as the counter and reference electrodes, respectively. A solution of 5-mM Fe(CN)6^4−^, 5-mM Fe(CN)6^3−^, and 0.1-M KCl in 100-mM MES (pH = 6.0) served as the electrolyte solution.

An SP-150 potentiostat (Bio-Logic, USA) was used for EIS analysis. EIS analysis was performed to distinguish between antibodies and antigens and Aβ(1–42) monomers and oligomers via the measurement of impedance differences. Figure 3 shows the schematic illustration of the experimental setup. The surface-modified nanostructured biosensor was used as the working electrode in electrochemical analysis, and Pt film and Ag/AgCl/3 M KCl functioned as the counter and reference electrodes, respectively. A solution of 5 mM Fe(CN)_6_^4−^, 5 mM Fe(CN)_6_^3−^, and 0.1 M KCl in 100 mM MES (pH = 6.0) was used as the electrolyte solution. The applied AC power amplitude was 10 mV. The scanning AC frequency was between 0.02 Hz and 200 kHz.

## 3 Results and Discussion

### 3.1 Western Blot and Fluorescence Analysis

**FIGURE 4:**
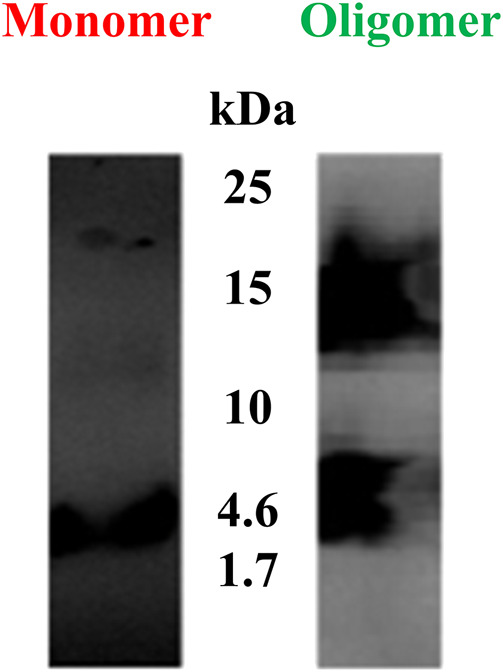
Western blots of Aβ(1–42) monomers and oligomers. The left lane shows a band of Aβ (1–42) monomers at ∼4.5 kDa. The right lane shows a wide band of Aβ(1–42) oligomers at ∼14.5 kDa.

In the present study, western blot analysis was performed to confirm the presence of Aβ(1–42) monomers and oligomers. Figure 4 indicates the different molar molecular weights of Aβ(1–42). The left lane shows a band of Aβ(1–42) monomers at ∼4.5 kDa. The right lane shows a wide band of Aβ(1–42) oligomers at ∼14.5 kDa. From the latter indicated result, it can be concluded that the Aβ(1–42) oligomer sample in the right lane contained Aβ(1–42) monomer, dimer, and oligomer conformations.

**FIGURE 5:**
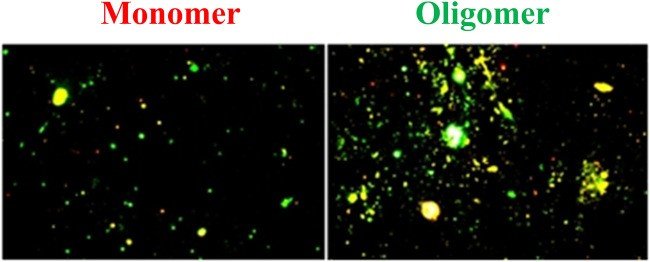
Fluorescence microscopic images of Aβ(1–42) monomers and oligomers. The coverage of bright spot of Aβ (1–42) monomers and oligomers are 10.31% and 27.53%, respectively.

The fluorescence microscopy images of 100-ng/ml Aβ(1–42) monomer and oligomer are shown in Figure 5. These images were analyzed using ImageJ ver. 1.53i to determine the coverage of bright spot. The results showed that the coverage of bright spot for the Aβ(1–42) monomer is 10.31% and the coverage of bright spot for oligomer are 27.53%. It was caused by Aβ (1–42) oligomer having more residual binding sites to capture 

### 3.2 Characterization of the Modified Biosensor

**FIGURE 6:**
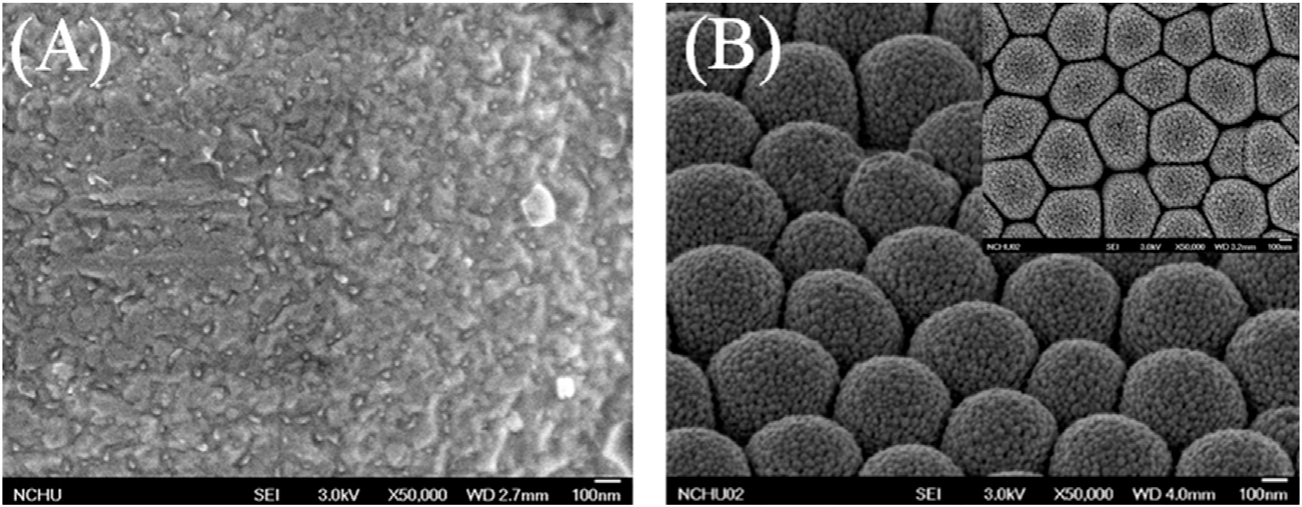
Scanning electron microscopic images of **(A)** the GNPs on a flat substrate, **(B)** the GNPs were uniformly deposited on the hemispheric nanostructure substrate.

To enhance the LOD and sensitivity of the biosensor, GNPs were deposited on thin Au film. Images captured using a scanning electron microscope and presented in Figure 6 show the comparison of flat and 3D nanostructure substrates with deposited GNPs. Au nanoparticles were aggregated on the flat substrate and dispersed on the nanostructured substrate. The GNPs on the flat substrate were aggregated to large particles because of the plate’s existing electrical field (Figure 6A). The GNPs were uniformly deposited on the hemispheric nanostructure substrate. The diameter of GNPs measured to be 10–15 nm (Figure 6B). The previous report proposed that the uniformly propagated electric flux perpendicular to the hemispheric thin Au film pulls the positive charges carrying Au nanoparticles in the electrolyte (Tsai et al., 2011). One of the most important factors to affect aggregation of GNPs on the nanostructured substrate is the deposition time. Precisely controlling the deposition time prevents the aggregation of GNPs. In this study, the deposited GNPs on the 3D nanostructure enlarged the overall surface area of the biosensor, resulting in an increased possibility of SAM and antibody attachment.

### 3.3 Impedance of Aβ(1–42)

**FIGURE 7:**
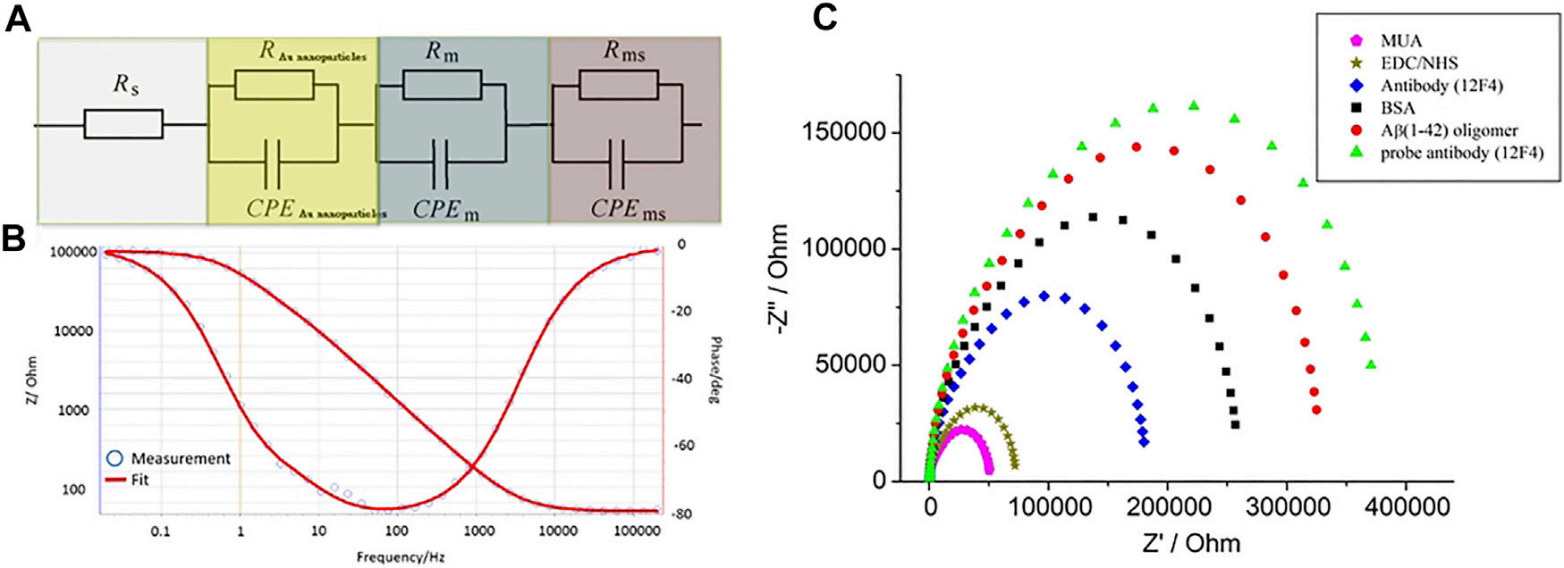
**(A)** Three parallel RC series were used to construct the equivalent circuit model, **(B)** the curves of experimental data were fitted by the equivalent circuit model. **(C)** The impedance plots of MUA, EDC/NHS, antibody, BSA, Aβ(1–42) oligomer 10 ng/ml, and probe antibody immobilized biosensors.

The charge transfer between the biosensor and the solution was measured by EIS. The deposited layer of GNPs altered the biosensor’s impedance and capacitance. Previous studies have shown that multiple layers deposited on gold electrodes cause an increase in impedance and a decrease in capacitance at low frequencies (Bogomolova et al., 2009; Darestani and Coster, 2012). Wu et al. proposed the inclusion of a specific circuit element to describe the relationship between different electrode interfaces (Wu et al., 2009). The combination of solution resistance (R_S_), Au nanoparticles’ layer resistance (R_Au nanoparticles_), modified layer resistance (R_m_), and resistance of the interface between the electrode and double layer (R_ms_) illustrates the total resistance of the biosensor. Due to the modified layer being rough and inhomogeneous, the capacitor can use the constant phase element (CPE) to describe such relationships. CPE was determined by Z(ω) = Q^−1^(jω)^−n^, where “Q” was equaled to capacitance and 0 < n < 1.When n = 1, CPE is almost a capacitor. The Au nanoparticle layer’s capacitance (CPE_nanoparticles_), modified layer capacitance (CPE_m_), and the capacitance of the interface between the electrode and electrical double layer (CPE_ms_) combined determine the total capacitance of biosensor. Three parallel RC series constructed the equivalent circuit model, which is presented in Figure 7A. The curves of experimental data were fitted by the equivalent circuit model, which we purposed for electrodes with BSA blocking, as shown in Figure 7B. All data were fitted in the equivalent circuit model for analysis. The surface-modified nanostructured biosensor was used as the working electrode in electrochemical analysis, and Pt film and Ag/AgCl/3M KCl functioned as the counter and reference electrodes, respectively. The EIS was measured with the electrolyte solution, containing 5-mM Fe(CN)6^4−^, 5-mM Fe(CN)6^3−^, and 0.1-M KCl in 100-mM MES (pH 6.0). Figure 7C shows the impedance plots of SAM molecules after immobilization. After the MUA, EDC/NHS, antibody, BSA, Aβ(1–42) oligomer 10 ng/ml, and probe antibody were immobilized, the values of R_ct_ were 51.2 ± 1.4, 73.4 ± 2.9, 180.83 ± 3.9, 259.7 ± 3.4, 323.1 ± 2, and 373.5 ± 2.7 kΩ, respectively. The fitted Rct values were increased after different stages of immobilization because MUA, EDC/NHS, antibody, BSA, and antigen have a higher electron transfer resistance as an insulating layer on the electrode interface. The differently increasing Rct values verified the fabrication process of the biosensor.

### 3.4 Optimization of Antibody Immobilization

**FIGURE 8:**
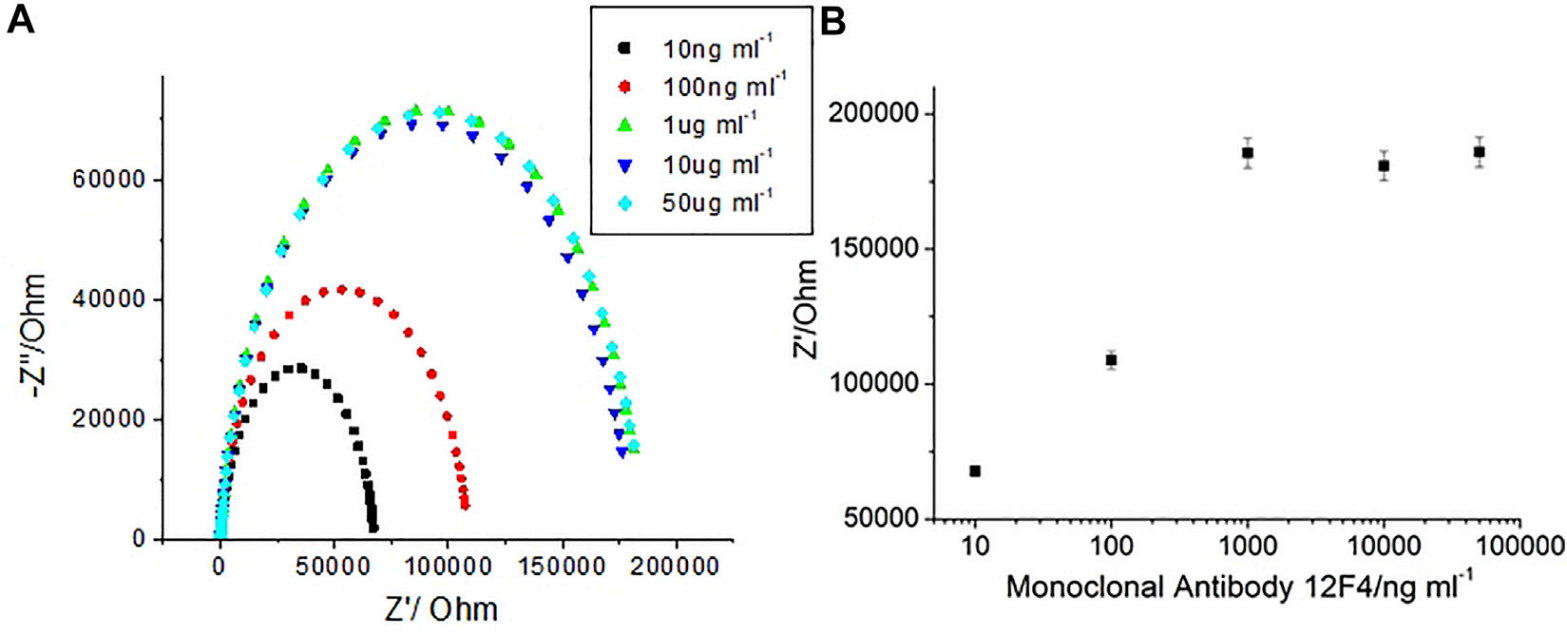
**(A)** EIS spetra with 12F4 antibodies at 1, 10, 100, 1, and 10 μg/ml, **(B)** The impedance with respect to corresponding antibody concentrations.

To evaluate the optimization of Aβ(1–42) capture, the antibody (12F4) stock solution was diluted into 1, 10, 100 ng/ml, 1 μg/ml, and 10 μg/ml solutions. The experimental data was fitted using an equivalent circuit model to estimate the resistance of the biosensor. The resistance estimates the quantity of antibodies (12F4) immobilized to the biosensor. The results presented in Figure 8 show that higher antibody (12F4) concentrations exhibit higher resistance. From the results in Table 1, it can be observed that the resistance of saturating antibodies (12F4) begins at a concentration of 100 ng/ml. Consequently, the tendency for changeable resistance levels indicates that the optimal concentration of immobilized antibodies (12F4) on the biosensor was ∼1 μg/ml.

**TABLE 1:** The impedance data corresponding antibody concentration.

	Antibody12F4	Antibody12F4	Antibody12F4	Antibody12F4	Antibody12F4
	(1 ng/ml)	(10 ng/ml)	(100 ng/ml)	(1 μg/ml)	(10 μg/ml)
${\text{R}}_{\text{total}}$ /kΩ	67.73 ± 2	108.91 ± 3.3	185.58 ± 5.6	180.83 ± 5.4	185.92 ± 5.6

### 3.5 Selectivity

**FIGURE 9:**
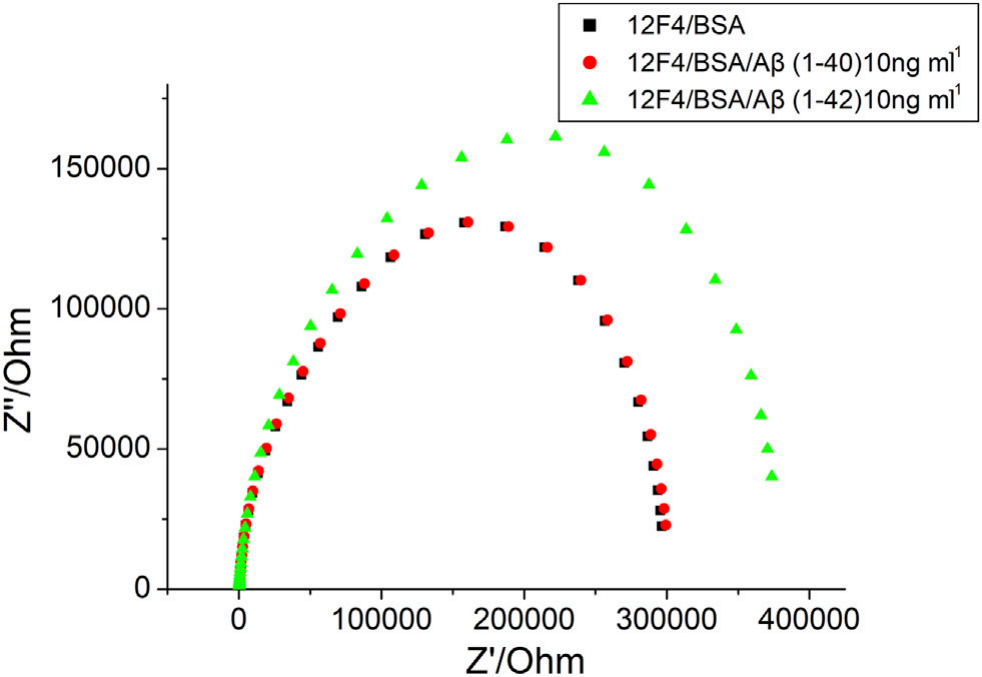
The EIS results of BSA, Aβ (1–40) 10 ng/ml and Aβ (1–42) 10 ng/ml, respectively.

For AD diagnosis, accurate Aβ (1–42) detection was needed. Selectivity of Aβ (1–42) a primary parameter. To investigate the selectivity of label-free experimentation, the biosensor was exposed to impedance analysis. Aβ(1–40) and Aβ(1–42) were immobilized at 10 ng/ml using monoclonal antibodies (12F4). The results presented in Figure 9 show that Aβ(1–42) impedance increased markedly, but no increase in Aβ(1–40) impedance post 1-h immobilization was observed. Therefore, the results suggest that the monoclonal antibody (12F4) shows a high specificity for Aβ(1–42). It was therefore concluded that the biosensor will accurately detect Aβ(1–42).

### 3.6 Impedance Analysis of Aβ(1–42) Monomers and Oligomers

**FIGURE 10:**
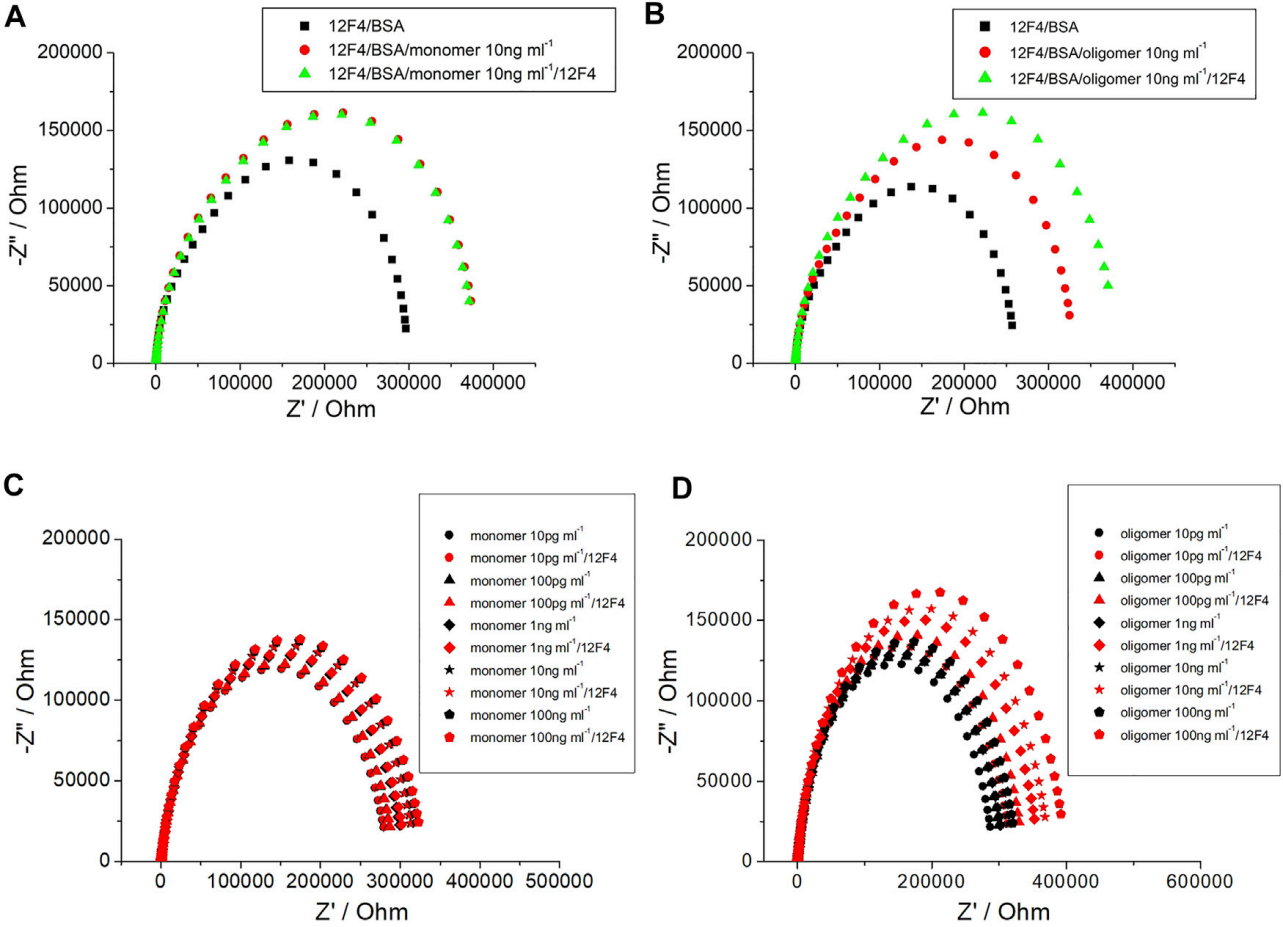
The EIS plot of **(A)** Aβ (1–42) monomer 10 ng/ml, **(B)** Aβ (1–42) oligomer 10 ng/ml. **(C)** Nyquist plots of Aβ (1–42) monomer and **(D)** Aβ (1–42) oligomer with different concentration of Aβ (1–42) at 10 pg/ml, 100 pg/ml, 1 ng/ml, 10 ng/ml and 100 ng/ml.

By determining impedance change, it can be suggested that the concentration of Aβ(1–42) captured by the biosensor may be qualitative. Furthermore, a method to detect Aβ(1–42) conformation was developed in the present study. After specific antibody (12F4) binding with Aβ(1–42), the same probe antibody (12F4) was applied to the biosensor, the biosensor was then subjected to incubation, and finally the impedance level was determined. When impedance was not markedly increased, as shown in Figure 10A, the Aβ(1–42) conformation was conducted the monomer. In contrast, the results showed that Aβ(1–42) conformation was conducted the oligomer, as shown in Figure 10B. The Aβ(1–42) monomer only has one binding site for antibody (12F4) binding, hence impedance does not increase when the same antibody (12F4) is immobilized on the biosensor again. Aβ(1–42) oligomers form due to the aggregation of many Aβ(1–42) monomers. If single Aβ(1–42) monomers that would otherwise compose Aβ(1–42) oligomers immobilize on the biosensor, it can be suggested that residual Aβ(1–42) monomers present additional binding sites that probe antibodies (12F4) may bind to. The Nyquist plots of Aβ(1–42) monomer with 10 pg/ml, 100 pg/ml, 1 ng/ml, 10 ng/ml, and 100 ng/ml is as shown in Figure 10C, while those of the Aβ(1–42) oligomer with 10 pg/ml, 100 pg/ml, 1 ng/ml, 10 ng/ml, and 100 ng/ml is as shown in Figure 10D. The impedance of Aβ(1–42) oligomer increase with respect to corresponding concentrations. The impedance data of Aβ(1–42) monomers and oligomers are shown in Tables 2 and 3. The resistance values for Aβ(1–42) monomers with 10 pg/ml, 100 pg/ml, 1 ng/ml, 10 ng/ml, and 100 ng/ml are 287.5 ± 4.6, 296 ± 4.2, 306 ± 3.7, 320.8 ± 4.6, and 329.1 ± 4.2 kΩ, respectively. The resistance values for 10 μg/ml 12F4 binding on Aβ (1–42) monomers with 10 pg/ml, 100 pg/ml, 1 ng/ml, 10 ng/ml, and 100 ng/ml are 291.2 ± 2.3, 297.3 ± 0.5, 309 ± 3.9, 324.4 ± 3.5, and 332.9 ± 4.6 kΩ, respectively. The resistance values for the Aβ(1–42) oligomer with 10 pg/ml, 100 pg/ml, 1 ng/ml, 10 ng/ml and 100 ng/ml are 298.6 ± 3.5, 306.3 ± 2.4, 313.5 ± 3.7, 323.1 ± 3.4, and 329.7 ± 3.1 kΩ, respectively. The resistance values for 10 μg/ml 12F4 binging on Aβ (1–42) oligomer with 10 pg/ml, 100 pg/ml, 1 ng/ml, 10 ng/ml, and 100 ng/ml are 317.8 ± 2.4, 338 ± 3.5, 361.1 ± 2.6, 373.5 ± and 2, 397.3 ± 2.7, respectively.

**TABLE 2:** The resistance values from experimental spectra for different Aβ (1–42) monomer and Aβ (1–42) oligomer concentrations. The statistical values of mean ± standard deviation were calculated in six repetitions.

	Monomer (10 pg/ml)	Monomer (100 pg/ml)	Monomer (1 ng/ml)	Monomer (10 ng/ml)	Monomer (100 ng/ml)
${\text{R}}_{\text{Aβ }\left(1-42\right)}$ /kΩ	287.5 ± 4.6	296 ± 4.2	306 ± 3.7	320.8 ± 4.6	329.1 ± 4.2
${\text{R}}_{12\text{F}4}$ /kΩ	291.2 ± 2.3	297.3 ± 0.5	309 ± 3.9	324.4 ± 3.5	332.9 ± 4.6
$\Delta {\text{R}}_{12\text{F}4}$ /kΩ	3.7	1.3	3	3.6	3.8
$P_{aggregation}$ /%	1.3	0.42	0.97	1.11	1.15
	**Oligomer (10 pg/ml)**	**Oligomer (100 pg/ml)**	**Oligomer (1 ng/ml)**	**Oligomer (10 ng/ml)**	**Oligomer (100 ng/ml)**
${\text{R}}_{\text{Aβ }\left(1-42\right)}$ /kΩ	298.6 ± 3.5	306.3 ± 2.4	313.5 ± 3.7	323.1 ± 3.4	329.7 ± 3.1
${\text{R}}_{12\text{F}4}$ /kΩ	317.8 ± 2.4	338 ± 3.5	361.1 ± 2.6	373.5 ± 2	397.3 ± 2.7
$\Delta {\text{R}}_{12\text{F}4}$ /kΩ	19.2	31.7	47.6	50.4	67.6
$P_{aggregation}$ /%	6.42	10.34	15.17	18.39	20.5

**TABLE 3:** Results of the detection of Aβ (1–42) oligomer concentrations in blood plasma using proposed biosensor by standard addition method.

Sample number	Added (ng/ml)	Found(ng/ml)	Recovery (%)	RSD (%, N = 3)
1	1	1.02 ± 0.04	102	3.9
2	10	10.3 ± 0.3	103	2.9
3	100	101.2 ± 1.5	101.2	1.5

$\Delta {\text{R}}_{12\text{F}4}$ refers to the amount of resistance changes for probe antibody (12F4) binding with Aβ(1–42). The $\Delta {\text{R}}_{12\text{F}4}$ value was calculated using the following equation:

$$\Delta {\text{R}}_{12\text{F}4}{=\text{R}}_{12\text{F}4}-{\text{R}}_{\text{Aβ }\left(1-42\right)}$$ (1)

where ${\text{R}}_{12\text{F}4}$ represents the sum resistance of impedance measured in same probe antibody (12F4) binding with Aβ(1–42) monomers or Aβ(1–42) oligomers in the last step, and ${\text{R}}_{\text{Aβ }\left(1-42\right)}$ represents the sum of resistance of impedance measured in the Aβ(1–42) immobilization step. The aggregation of Aβ(1–42) was determined via the following equation:

Paggregation=ΔR12F4RAβ(1−42)×100% (2)

where $P_{aggregation}$ represents the aggregation percentage: the value increase when more monomers aggregate and exhibit oligomer conformation.

**FIGURE 11:**
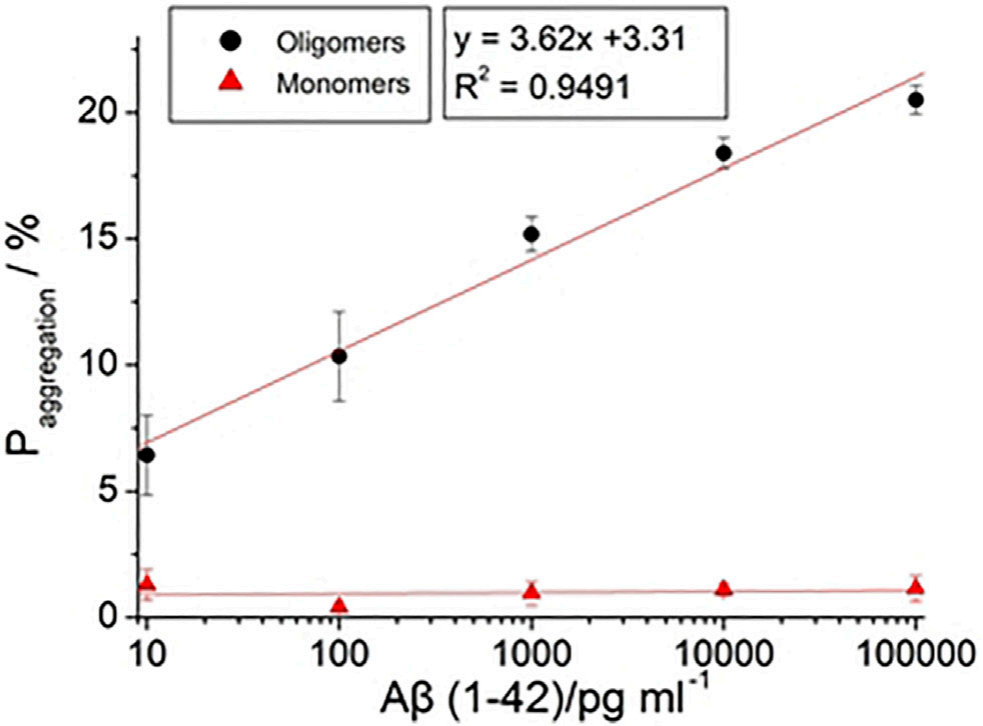
The calibration curve of $P_{aggregation}$ with different concentration of Aβ (1–42) with 10 pg/ml, 100 pg/ml, 1 ng/ml, 10 ng/ml, and 100 ng/ml.

The impedance data of Aβ(1–42) monomers and oligomers are shown in Table 2. $P_{aggregation}$ of Aβ(1–42) monomers were 1.65%, 1.03%, 0.46%, 0.66%, and 0.11% from 10 pg/ml, 100 pg/ml, 1 ng/ml, 10 ng/ml, and 100 ng/ml, respectively. $P_{aggregation}$ did not increase with Aβ(1–42) monomers concentration. Conversely, $P_{aggregation}$ of Aβ(1–42) oligomers were 6.42%, 10.34%, 15.17%, 18.39%, and 20.5% from 10 pg/ml, 100 pg/ml, 1 ng/ml, 10 ng/ml, and 100 ng/ml, respectively. $P_{aggregation}$ of Aβ(1–42) oligomers increased significantly with concentration of Aβ(1–42) oligomers. The plot in Figure 11 shows the individual aggregation percentages of Aβ(1–42) monomers and Aβ(1–42) oligomers. The plot illustrating the aggregation of Aβ(1–42) monomers approached a plateau regardless of the concentration increasing from 10 pg/ml to 100 ng/ml. In clinical pathology, the existence of Aβ(1–42) oligomers is more important than Aβ(1–42) monomers. The plot detailing the aggregation percentages of Aβ(1–42) oligomers resulted in the development of the following equation: $P_{aggregation}$ = {3.62 × log[Aβ(1–42)]+3.31}  ×  100% with an R^2^ value of 0.9491, where [Aβ(1–42)] is the concentration of Aβ(1–42). The level of linear detection ranged from 10 pg/ml to 100 ng/ml. The LOD value was calculated based on the LOD equation = 3.3σ/S, where σ is the standard deviation of the response, S is the slope of the calibration curve. The LOD value of the biosensor was 113 fg/ml. The impedance measuring results of linearity (R^2^=0.9491) extended from 10 pg/ml to 100 ng/ml, which was a much greater range and lower LOD than previous studies employing the ELISA method. To demonstrate the practical performances of the developed biosensor, the Aβ(1–42) oligomer was analyzed in real samples of 10 fold-diluted healthy human blood plasma by standard addition method, the results is as listed in Table 3. We observed the found concentrations of Aβ (1–42) oligomer is 1.02 ± 0.04 ng/ml, 10.3 ± 0.3 ng/ml and 101.2 ± 1.5 ng/ml for added 1 ng/ml, 10 ng/ml, and 100 ng/ml, respectively. The recovery is 102%, 103%, and 101.2% for added 1, 10, and 100 ng/ml, respectively. The developed biosensor has low interference, indicating that the biosensor represents a suitable platform for the detection of Aβ (1–42) oligomer in blood plasma of AD patients. The range of detectable Aβ(1–42) concentrations and the recovery in real samples were adequate to allow for differentiation of Aβ(1–42) conformations in future clinical use. The features of the biosensor observed in this study can be attributed to the hypothesis stated in the report proposed. GNPs can be deposited in a uniform manner on the 3D nanostructure of the PC template sputtered thin Au film via electrochemical methods. This method enhanced the surface area of the biosensor, meaning that more SAM molecules were able to assemble on the biosensor. Therefore, the biosensor exhibited enhanced biomarker capture ability. Finally, the employed impedance analysis was a label-free technique, hence signals received by the electrical circuit amplifier prevented optical restrictions.

### 3.7 Repeatability, Reproducibility, and Stability

The mean standard deviation was calculated in six repetitions. The relative standard deviations (RSD) of the R_12F4_ value calculated from Table 2 for different Aβ (1–42) oligomer concentrations were 0.12%, 0.25%, 0.72%, 0.12%, and 0.68%, respectively. These results represent that the developed biosensor had good repeatability.

To investigate the reproducibility, 30 nanostructured immunoassay biosensors were prepared under the same conditions and were used to determine 1 ng/ml Aβ(1–42) oligomer on different days with different immunosensors prepared for each test. The aggregation value was used to determine the sensor’s reproducibility in this study. The RSD was 5.2% for the 30 different sandwich immunoassay biosensors in Supplementary Table S1, therefore demonstrating the reproducibility of the proposed immunoassay biosensors.

**FIGURE 12:**
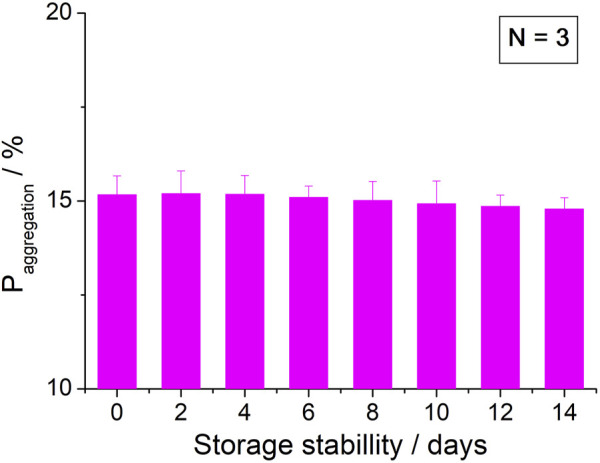
Stability of the biosensor for 2 weeks.

To investigate the stability, nanostructured immunoassay biosensors were stored in a refrigerator at 4°C after BSA immobilization. Before testing the stability, the biosensors were replaced in room temperature for 10 min. The stability of the nanostructured immunoassay biosensors was estimated by determining 1 ng/ml Aβ (1–42) oligomer for 0, 2, 4, 6, 8, 10, 12, and 14 d. The aggregation value was determined using the sensor’s stability in this study. The $P_{aggregation}$ were 15.17, 15.2, 15.18, 15.1, 15.02, 14.93, 14.86, and 14.79 for 0, 2, 4, 6, 8, 10, 12, and 14 d, respectively, as shown in Figure 12. After 2-week storage, the $P_{aggregation}$ was slightly reduced to around 2.5%. The above results show that the biosensors have acceptable repeatability, reproducibility, and stability, which was compared to the reported sensors.

## 4 Conclusion

We developed a sensitive biosensor for the detection of Aβ(1–42). To enhance the sensitivity of detection, a thin Au film with GNPs was deposited on the 3D nanostructure PC substrate in a uniform manner. Non-aggregated GNPs enhanced the possibility of SAM molecules binding to the biosensor. Additionally, a electrochemical sandwich assay was performed in the present study. Monoclonal antibodies (12F4) were immobilized on the biosensor to capture Aβ(1–42), and the biosensor was again incubated with probe antibodies (12F4) to allow them to bind to residual Aβ(1–42) binding sites. The results of western blot and fluorescence analysis confirm previous reports of differing Aβ(1–42) conformations. An EIS analysis was implemented to determine the impedance of the biosensor, allowing for the differentiation between Aβ(1–40), Aβ(1–42) monomer, and Aβ(1–42) oligomer compositions. The EIS results show that the impedance of Aβ(1–40) does not increase or decrease with the concentration of Aβ(1–40), which suggests that the biosensor exhibits good selectivity for Aβ(1–42). The Aβ(1–42) monomer has one binding site bound by an antibody, hence why the impedance did not change despite changing concentrations. The impedance of Aβ(1–42) oligomers steadily increases with concentration. The linear detection range of Aβ(1–42) oligomers ranged between 10 pg/ml and 100 ng/ml. The LOD values for Aβ(1–42) oligomers can be estimated to be 113 fg/ml. Compared with ELISA and western blot analyses, the determination of Aβ(1–42) concentration using the proposed nanostructured biosensor has wide range of detection, requires a low sample volume (30 μl), a short preparation time (1.5 h), and a short detection time (2 min).

## Data Availability

The raw data supporting the conclusion of this article will be made available by the authors, without undue reservation.
