# Supplementary material for: Detection of Amyloid-β(1–42) Aggregation With a Nanostructured Electrochemical Sandwich Immunoassay Biosensor
Source: Front Bioeng Biotechnol. 2022 Mar 16;10:853947. doi: 10.3389/fbioe.2022.853947 (PMC8965719; doi:10.3389/fbioe.2022.853947)
Supplement: Supplementary file 1 [file DataSheet1.PDF]

TABLE 1 | The impedance data corresponding Antibody concentration.

	Antibody12F4 (1ng/ml)	Antibody12F4 (10ng/ml)	Antibody12F4 (100ng/ml)	Antibody12F4 (1μg/ml)	Antibody12F4 (10μg/ml)
R_{total}/kΩ	67.73±2.4	108.91±3.7	185.58±5.6	180.83±5.2	185.92±5.3

TABLE 2 | The resistance values from experimental spectra for different Aβ (1-42) monomer and Aβ (1-42) oligomer concentrations. The statistical values of mean ± standard deviation were calculated in six repetitions

	Monomer (10pg/ml)	Monomer (100pg/ml)	Monomer (1ng/ml)	Monomer (10ng/ml)	Monomer (100ng/ml)
$R_{A\beta\ (1-42)}/k\Omega$	287.5±4.6	296±4.2	306±3.7	320.8±4.6	329.1±4.2
$R_{12F4}/k\Omega$	291.2±2.3	297.3±0.5	309±3.9	324.4±3.5	332.9±4.6
$\Delta R_{12F4}/\ k\Omega$	3.7	1.3	3	3.6	3.8
$P_{aggregation}/\%$	1.3	0.42	0.97	1.11	1.15
	Oligomer (10pg/ml)	Oligomer (100pg/ml)	Oligomer (1ng/ml)	Oligomer (10ng/ml)	Oligomer (100ng/ml)
$R_{A\beta\ (1-42)}/k\Omega$	298.6±3.5	306.3±2.4	313.5±3.7	323.1±3.4	329.7±3.1
$R_{12F4}/k\Omega$	317.8±2.4	338±3.5	361.1±2.6	373.5±2	397.3±2.7
$\Delta R_{12F4}/\ k\Omega$	19.2	31.7	47.6	50.4	67.6
$P_{aggregation}/\%$	6.42	10.34	15.17	18.39	20.5

TABLE 3 | Results of the detection of Aβ (1-42) oligomer concentrations in blood plasma using proposed biosensor by standard addition method

Sample number	Added (ng/ml)	Found(ng/ml)	Recovery (%)	RSD (%, N = 3)
1	1	1.02±0.04	102	3.9
2	10	10.3±0.3	103	2.9
3	100	101.2±1.5	101.2	1.5

TABLE S1 | The resistance values of 30 different sandwich immunoassay biosensors for reproducibility study

Biosensor No.	$R_{A\beta\ (1-42)}/k\Omega$	$R_{12F4}/k\Omega$	$\Delta R_{12F4}/\ k\Omega$	$P_{aggregation}/\%$
1	314.47	366.83	52.36	16.65
2	315.07	363.15	48.08	15.26
3	325.22	369.97	44.75	13.76
4	292.98	337.25	44.27	15.11
5	308.12	354.77	46.65	15.14
6	338.09	385.22	47.13	13.94
7	295.27	341.45	46.18	15.64
8	290.39	337.52	47.13	16.23
9	352.64	402.15	49.51	14.04
10	291.63	335.90	44.27	15.18
11	329.03	379.01	49.98	15.19
12	336.68	387.62	50.94	15.13
13	274.54	319.29	44.75	16.3
14	303.86	349.56	45.7	15.04
15	301.71	351.22	49.51	16.41
16	364.91	416.80	51.89	14.22
17	313.08	356.88	43.8	13.99
18	331.02	379.58	48.56	14.67
19	328.75	378.26	49.51	15.06
20	308.74	355.39	46.65	15.11
21	334.75	384.26	49.51	14.79
22	304.10	349.32	45.22	14.87
23	307.64	354.77	47.13	15.32
24	336.45	387.86	51.41	15.28
25	300.74	345.49	44.75	14.88
26	269.37	313.17	43.8	16.26
27	323.70	370.83	47.13	14.56
28	326.20	371.90	45.7	14.01
29	301.27	348.87	47.6	15.8
30	326.22	372.87	46.65	14.3
Average	314.89±21.6	362.24±23.34	47.35±2.49	15.07±0.78
RSD (%)	6.86	6.44	5.26	5.2
